# Early use of high-efficacy disease‑modifying therapies makes the difference in people with multiple sclerosis: an expert opinion

**DOI:** 10.1007/s00415-022-11193-w

**Published:** 2022-05-24

**Authors:** Massimo Filippi, Maria Pia Amato, Diego Centonze, Paolo Gallo, Claudio Gasperini, Matilde Inglese, Francesco Patti, Carlo Pozzilli, Paolo Preziosa, Maria Trojano

**Affiliations:** 1 Neuroimaging Research Unit, Division of Neuroscience, IRCCS San Raffaele Scientific Institute, Via Olgettina, 60, 20132 Milan, Italy; 2 Neurology Unit, IRCCS San Raffaele Scientific Institute, Via Olgettina, 60, 20132 Milan, Italy; 3 Neurorehabilitation Unit, IRCCS San Raffaele Scientific Institute, Via Olgettina, 60, 20132 Milan, Italy; 4 Neurophysiology Service, IRCCS San Raffaele Scientific Institute, Via Olgettina, 60, 20132 Milan, Italy; 5 Vita-Salute San Raffaele University, Milan, Italy; 6 Department NEUROFARBA, University of Florence, Florence, Italy; 7 IRCCS Fondazione Don Carlo Gnocchi, Florence, Italy; 8 Department of Systems Medicine, Tor Vergata University, Rome, Italy; 9 Unit of Neurology, IRCCS Neuromed, Pozzilli, IS Italy; 10 Department of Neuroscience, University of Padova, Padua, Italy; 11 Department of Neurosciences, S Camillo Forlanini Hospital Rome, Rome, Italy; 12 Department of Neuroscience, Rehabilitation, Ophthalmology, Genetics, Maternal and Child Health (DINOGMI), University of Genoa, Genoa, Italy; 13 IRCCS Ospedale Policlinico San Martino, Genoa, Italy; 14 Department GF Ingrassia, Medical, Surgical Science and Advanced Technologies, University of Catania, Catania, Italy; 15 Center for Multiple Sclerosis, Policlinico “G Rodolico”, University of Catania, Catania, Italy; 16 S. Andrea MS Center, Sapienza University, Rome, Italy; 17 Department of Basic Medical Sciences, Neuroscience, and Sense Organs, University of Bari “Aldo Moro”, Bari, Italy

**Keywords:** Multiple sclerosis, Disease-modifying drugs, Disease progression

## Abstract

Multiple sclerosis (MS) is a chronic and progressive neurological disease that is characterized by neuroinflammation, demyelination and neurodegeneration occurring from the earliest phases of the disease and that may be underestimated. MS patients accumulate disability through relapse-associated worsening or progression independent of relapse activity. Early intervention with high-efficacy disease-modifying therapies (HE-DMTs) may represent the best window of opportunity to delay irreversible central nervous system damage and MS-related disability progression by hindering underlying heterogeneous pathophysiological processes contributing to disability progression. In line with this, growing evidence suggests that early use of HE-DMTs is associated with a significant greater reduction not only of inflammatory activity (clinical relapses and new lesion formation at magnetic resonance imaging) but also of disease progression, in terms of accumulation of irreversible clinical disability and neurodegeneration compared to delayed HE-DMT use or escalation strategy. These beneficial effects seem to be associated with acceptable long-term safety risks, thus configuring this treatment approach as that with the most positive benefit/risk profile. Accordingly, it should be mandatory to treat people with MS early with HE-DMTs in case of prognostic factors suggestive of aggressive disease, and it may be advisable to offer an HE-DMT to MS patients early after diagnosis, taking into account drug safety profile, disease severity, clinical and/or radiological activity, and patient-related factors, including possible comorbidities, family planning, and patients’ preference in agreement with the EAN/ECTRIMS and AAN guidelines. Barriers for an early use of HE-DMTs include concerns for long-term safety, challenges in the management of treatment initiation and monitoring, negative MS patients’ preferences, restricted access to HE-DMTs according to guidelines and regulatory rules, and sustainability. However, these barriers do not apply to each HE-DMT and none of these appear insuperable.

## Introduction

Multiple sclerosis (MS) is a chronic and progressive neurological disease of the central nervous system (CNS) characterized by heterogeneous clinical manifestations and disease course [1]. Pathologically, MS is typified by inflammation, demyelination and neurodegenerative phenomena that occur from the earliest phases of the disease and that may be subclinical and underestimated at the beginning [1].

Specific disease-modifying therapies (DMTs) are currently available to prevent the accumulation of MS-related structural brain damage and its detrimental effects for MS patients [2–8]. During the last years, the landscape of MS treatment has substantially evolved thanks to the introduction of more and more effective DMTs [2–8]. Based on their efficacy, currently available DMTs are commonly distinguished as moderate-efficacy (ME) DMTs (glatiramer acetate, interferon-beta [IFN-β], teriflunomide and dimethyl fumarate) and high-efficacy (HE) DMTs (natalizumab, fingolimod, ozanimod, siponimod, alemtuzumab, cladribine, ocrelizumab, and ofatumumab) [2–8].

Treatment decision-making among these different DMTs is typically influenced by several aspects, including MS patients’ profile (demographic variables, clinical features, clinical, biological and neuroradiological prognostic factors, presence of comorbidities, patients’ preference and patients’ lifestyle), guidelines currently available [4, 5], limited access to specific DMTs, due to restrictions on the approved regulatory label population imposed by reimbursement bodies, and safety concerns.

Recent growing pieces of evidence are suggesting that early initiation of HE-DMTs may have a beneficial long-term impact on disease progression in MS patients [9–15], thus underlying the need for offering an early treatment with an HE-DMT to MS patients.

In this Expert Opinion paper, we reported the conclusions of the meeting held in Rome, Italy, on the 1st of December 2021, which included Italian experts in the field of MS treatment and management. Specifically, clinically relevant statements regarding the early use of HE-DMTs for MS patients have been defined as an agreement among the experts (Table 1) and have been better clarified in the different sections of this manuscript.

**Table 1:** Summary of the agreed statements on the early use of HE-DMTs in people with MS

Topic	Agreed statements
Therapeutic goals in MS	MS is a chronic and progressive neurological disease Early progression in MS is characterized by neuroinflammation and subclinical neurodegeneration that may be underestimated Early disease phases are the best window of therapeutic opportunity in MS Treatment goals consist in hindering the underlying pathophysiological mechanisms (i.e., inflammation and neurodegeneration) early in the disease course preventing the progression of irreversible disability
Best treatment strategy to reach the therapeutic goals	A higher benefit could be reached with an early initiation of an HE-DMT, irrespective of prognostic factors Early initiation of an HE-DMT could be associated with a better risk/benefit ratio vs an escalation approach (which is associated with a lack of disease control)
HE-DMTs: defining high efficacy and supporting evidence for their early use	A therapy can be defined as HE-DMT if a therapeutic effect can be proven on ≥ 1 outcome of inflammation Substantial decrease of annualized relapse rate and/or Substantial decrease of MRI activity (new/enlarging T2-hyperintense lesions and/or Gd-enhancing lesions) AND ≥ 1 outcome of disease progression: Substantial decrease of clinical disability progression: confirmed worsening of EDSS score and its functional system scores, cognitive deterioration, composite scores (e.g., MSFC, EDSS worsening plus ≥ 20% minimum threshold change for T25FWT and 9HPT) Substantial effect on MRI measures of neurodegeneration: global or regional brain and spinal cord atrophy Substantial effect on body fluid biomarkers: neurofilament light chain levels PROs
Treatment strategies based on patients’ profiles	It is advisable to offer an early treatment with an HE-DMT to all MS patients It is mandatory to offer early treatment initiation with an HE-DMT in case prognostic factors are indicative of aggressive disease In evaluating treatment options, patient-related factors should be considered (e.g., comorbidities, preferences, family planning, etc.)
Overcoming barriers to HE-DMTs’ early use	Equal access to care should be guaranteed to all MS patients (i.e., access to highly specialized MS Centres, with experienced Neurologists) who should receive an appropriate treatment Barriers to HE-DMTs’ early use may exist, which include: Perception of an unfavorable risk/benefit ratio in the long term Challenges in medication therapy management Negative patients’ preferences Sustainability These barriers do not apply to each DMT and none of these are insuperable There are no signs of concerns on long-term risk/benefit ratio There are no logistics or therapy management issues (even if some differences between therapies exist, such as in terms of administration and follow-up/monitoring patterns)

*9HPT* Nine-Hole Peg Test, *EDSS* Expanded Disability Status Scale, *Gd* gadolinium, *HE*-*DMT* high-efficacy disease-modifying therapy, *MRI* magnetic resonance imaging, *MS* multiple sclerosis, *MSFC* multiple sclerosis functional composite, *PROs* patient’s reported outcomes, *T25FWT* timed 25-foot walk test

## Therapeutic goals in MS

MS progression starts early, but may be underestimated in the earliest phases of the disease (Table 1) [16–18]. Treatment strategies for MS aim to hinder the underlying pathophysiological mechanisms early in the disease course preventing the progression of irreversible disability.

The prevention of overt demyelination and inflammation, with a substantial reduction of clinical and magnetic resonance imaging (MRI) disease activity (i.e., number and severity of clinical relapses, new/enlarging T2-hyperintense lesions and gadolinium-enhancing lesions) is a relevant therapeutic goal in MS [19]. Results from randomized controlled trials (RCTs) and real-world observational studies have consistently highlighted the combined beneficial effects of most of the available DMTs in reducing clinical relapses and new lesion formation at MRI [3, 6–8].

However, the mainstay of treatment goals for MS is moving towards the prevention of neurodegenerative phenomena, the slowing of irreversible disease progression, and neuroprotection [2, 7, 19, 20].

Growing evidence is suggesting that disability progression in MS patients is only partially secondary to the occurrence of new focal inflammatory demyelinating lesions and clinical relapses (i.e., relapse-associated worsening), whereas progression independent of relapse activity (PIRA) starts from the biological onset of MS and becomes the principal and most relevant driver of disability accumulation in the progressive forms of MS [21–23].

Neuro-axonal loss is thought to represent the major contributor to irreversible clinical disability in MS patients [17, 18, 24, 25]. In the presence of acute, but also chronic and compartmentalized, inflammation, direct immune-mediated damage, mitochondrial and metabolic dysfunctions, oxidative damage from iron deposition and microglial activation, and excitatory/inhibitory imbalance may cause a gradual and progressive neuro-axonal damage [17, 18, 24, 25]. Although such pathological processes occur from the beginning of the disease, the accumulation of structural CNS damage typically remains subclinical and hardly detectable in the earliest phases of MS due to CNS plasticity. This phenomenon reflects the ability of the CNS to change and modulate its activity in response to pathological stimuli and damage by reorganizing its structure, functions, or connections [26, 27]. Structural and functional CNS plasticity may be able, especially in younger MS patients, with milder structural damage and disease duration, to compensate the progressive accumulation of MS-related structural damage. However, after a certain threshold, such ‘brain reserve’ is no more able to guarantee a preservation of functions, thus determining a clinically detectable disability progression. Such compensatory mechanisms have profound implications and should be taken into account in the treatment paradigm for MS patients. If the detrimental effects of MS in terms of disability progression become evident only after a substantial and irreversible CNS damage has occurred, the use of HE-DMTs only in the most advanced and severe phases of the disease strongly limits our therapeutic window of opportunity and the efficacy of DMTs to prevent further neurodegeneration and exert beneficial effects for MS patients.

## Best treatment strategy to reach the therapeutic goals

In the last years, the increasing number of approved DMTs is allowing to tailor therapy according to individual patients’ needs, with regard to efficacy, safety aspects and patients’ preferences.

Currently, two different treatment algorithms are typically used in the clinical scenario. A first approach is defined as ‘escalation therapy’ and is more focused on safety. It suggests to start with lower-risk ME-DMTs, which are generally moderately effective, but well tolerated and with limited side effects and safety concerns [4, 5]. In case of side effects or poor compliance, another ME-DMT is indicated, whereas in case of inefficacy (i.e., new clinical relapses and/or MRI activity), escalation to an HE-DMT should be considered [4, 5].

The second treatment approach, known as ‘induction therapy’, prioritizes efficacy and is based on an early and aggressive intervention on the immune system to better prevent the accumulation of irreversible CNS damage and clinical disability, especially in MS patients with negative prognostic factors [4, 5].

Despite the availability of specific consensus guidelines to help neurologists in treatment decision-making [4, 5], a standardized approach still needs to be defined. However, the timing of HE-DMT start has substantial implication and long-term impact on clinical disability and disease progression in MS patients, thus questioning the rationale of the escalation strategy and supporting a wider use of HE-DMTs already from the earliest phases of the disease [9–15].

As discussed later, an early initiation of an HE-DMT may promote a higher benefit, irrespective of negative prognostic factors and may be associated with a better risk/benefit ratio compared to an escalation approach (which is more frequently associated with a lack of disease control) (Table 1).

## HE-DMTs: defining high efficacy

Despite the introduction of DMTs that have been proven to be more and more effective, a definition for HE-DMT is still lacking [7]. The neurologists of this Expert Opinion paper agreed that a drug should be defined as HE-DMT if its substantial therapeutic effect can be proven on ≥ 1 outcome of inflammation/demyelination but also on ≥ 1 outcome of disease progression (Table 1).

Measures of inflammation and demyelination include the annualized relapse rate (ARR)[19] and MRI measures of disease activity (new/enlarging T2-hyperintense lesions and/or Gd-enhancing lesions), which represent surrogate markers of disease activity [19, 28, 29].

**Fig. 1:**
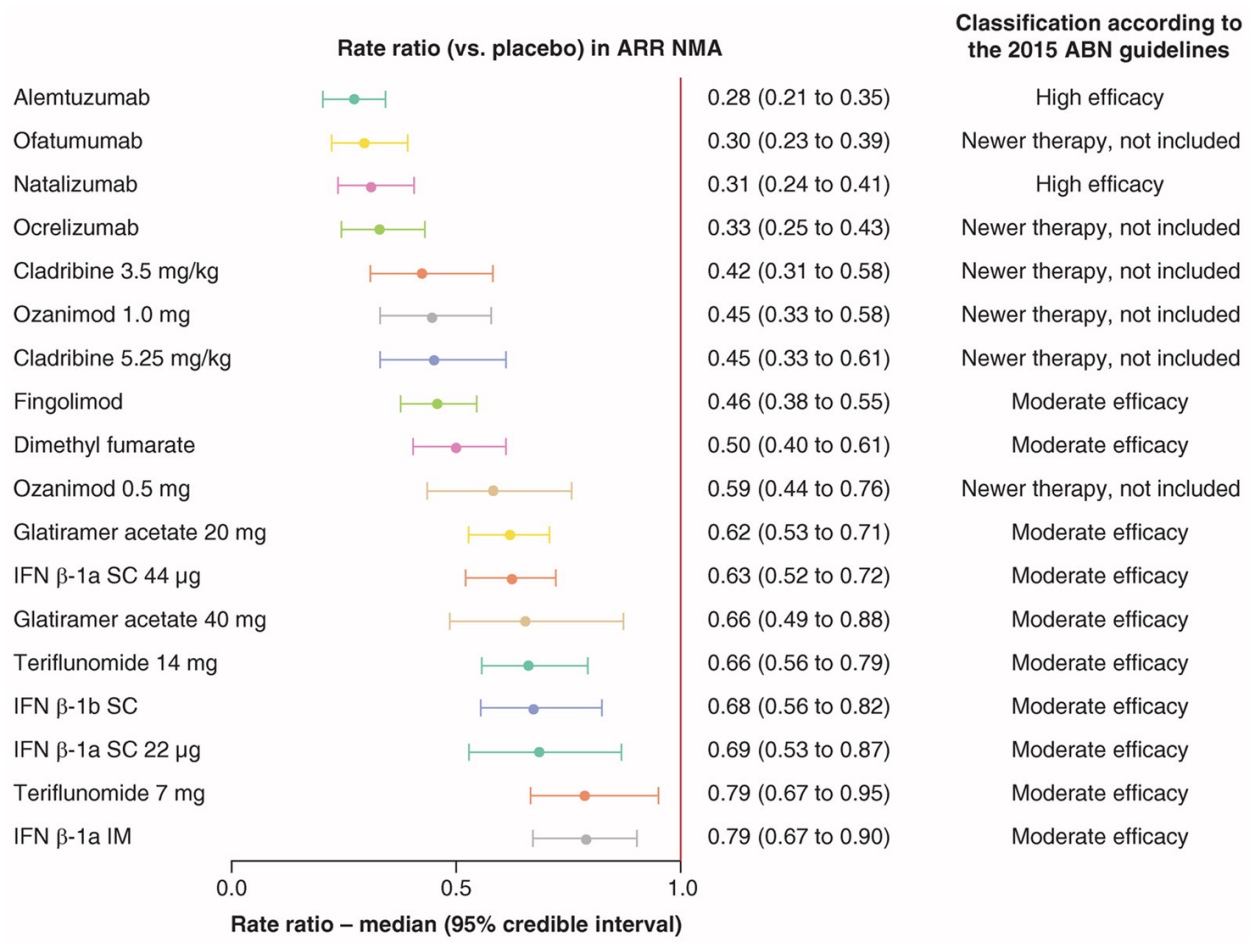
Annualized relapse rate of each DMT relative to placebo. Annualized relapse rate network meta-analysis forest plot (versus placebo) with efficacy class for each disease-modifying therapies (2015 Association of British Neurologists guidelines). Rate ratios from the ARR NMA may not directly align with the relapse rate reduction values used by the ABN to group the DMTs. The ABN guidelines were published in 2015, so the NMA estimates were informed by additional more recently published trials. *ABN* Association of British Neurologists, *ARR* annualized relapse rate, *DMT* disease-modifying therapy, *IFN* interferon, *IM* intramuscular, *NMA* network meta-analysis, *SC* subcutaneous. Reproduced from Samjoo IA, Worthington E, Drudge C, Zhao M, Cameron C, Häring DA, Stoneman D, Klotz L, Adlard N. Efficacy classification of modern therapies in multiple sclerosis. J Comp Eff Res 2021; 10(6): 495–507. https://doi.org/10.2217/cer-2020-0267. An open-access article under the Attribution-NonCommercial-NoDerivatives 4.0 Unported License

According to the results from RCTs and observational studies (see [3, 8] for comprehensive meta-analyses), HE-DMTs should determine a substantial decrease of the ARR. Experts suggest to consider as HE-DMTs those treatments having an average reduction of ARR at least 50% more than placebo (Fig. 1) [3, 8]. Since MRI measures are much more sensitive than clinical evaluation in detecting disease activity and results from RCTs consistently demonstrated a strong suppression of MRI activity of several DMTs, a reduction ≥ 70% compared to placebo is likely to be considered relevant according to the Experts’ opinion [19, 29].

Beside a strong anti-inflammatory activity, an HE-DMT should also limit disability progression. Clinically, the evidence of beneficial effects of DMTs on MS progression is based on the demonstration of a significant reduction of EDSS score worsening (see [3] for a comprehensive meta-analysis). Experts suggest to consider HE-DMTs those treatments having an average reduction of disability progression at least 30% more than placebo [3]. However, the EDSS score has intrinsic limitations [30–32], thus recent proposals have moved towards a more specific definition [31], by creating composite scores integrating quantitative performance measures of locomotor functions and cognition (e.g., EDSS plus ≥ 20% minimum threshold change for Timed 25-Foot Walk test [walking ability], 9-Hole Peg Test [hand dexterity] and Paced Auditory Serial Addition Test or Symbol Digit Modalities Test [cognitive performances]) [33–36] to evaluate disability progression more comprehensively.

In addition to preventing disability progression, sustained disability improvement, defined as an EDSS decrease of one point if the baseline EDSS score was ≤ 5.0 and of a half-point if the baseline EDSS score was ≥ 5.5 [35], may represent an additional relevant endpoint supporting the beneficial effects of HE-DMTs [37–43].

The effect on cognitive decline (e.g., ≥ 4-point decrease in SDMT score) [44] is also receiving more and more attention as a clinically relevant measure to identify MS progression. However, the beneficial effects of HE-DMTs on this parameter still need to be fully explored [45, 46].

Similarly, patient-reported outcomes (PROs) may offer a more adequate assessment of the impact of the disease on the daily life of MS patients [47], but the positive effects of HE-DMTs has been only partially explored [48].

Despite this, the clinical evaluation of disease progression is only partially connected with the underlying pathobiological changes, which begin earlier and progress through the disease course in a continuum pattern [49, 50]. Accordingly, biological markers that are more sensitive and specific to neurodegeneration processes may be useful to better detect the beneficial effects of HE-DMTs. These include the quantification of global and regional brain and spinal cord atrophy using MRI, which represents a surrogate marker of clinical disability progression [19, 28, 51–54], and blood levels of neurofilament light chains [55–57]. The treatment effect on brain atrophy has been found to correlate with clinical disability progression and this effect seems to be independent from the effect of active MRI lesions [54]. An annual threshold of brain volume loss of -0.40% has also been proposed to best discriminate brain atrophy in MS patients vs HC [58], and, possibly, to demonstrate the beneficial effects of HE-DMTs [59].

Similarly, findings supporting the beneficial effects of HE-DMTs in reducing neurofilament light chain levels are growing [57], even though their ability to predict subsequent clinical and MRI outcomes still needs to be fully demonstrated [56].

## HE-DMTs: supporting evidence for their early use

The rationale to start early HE-DMTs to limit disease progression from the earliest phases of the disease is supported by a growing number of studies evaluating large cohorts of MS patients and showing consistently that early initiation of HE-DMTs compared to ME-DMTs or early switch to HE-DMTs may achieve the maximum benefit on MS disease evolution, due to their higher ability in limiting more effectively the accumulation of irreversible clinical disability, the evolution to secondary progressive MS and the progression of brain atrophy (Table 2) [9–15].

**Table 2:** Summary of the main studies supporting the beneficial effects of early use of HE-DMTs in MS patients

Study	Study design	Comparisons of treatment strategy(ies)	MS patients	Main findings
Harding et al. [9]	Retrospective single-center data from the southeast Wales	EIT vs ESC *ESC: IFN-β, GA, DMF, FTY, TERI* *EIT: NAT, ALEM*	EIT: *n* = 104 ESC = *n* = 488	5-year change in EDSS score was lower in the EIT vs ESC (mean [SD] = 0.3 [1.5] vs 1.2 [1.5], *p* < 0.001), which remained significant after adjustment for relevant covariates (*β* = − 0.85; 95% CI − 1.38; − 0.32, * p* = 0.002) Median time to sustained accumulation of disability was 6.0 (95% CI 3.17; 9.16) years for EIT and 3.14 (2.77; 4.00) years for ESC (*p* = 0.05)
Brown et al. [10]	Retrospective data from the MSBase registry	HE-DMT vs ME-DMT Early vs late escalation from ME-DMT to HE-DMT (≤ 5 vs > 5 years) *ME-DMT: IFN-β, GA* *HE-DMT: FTY, NAT, ALEM*	ME-DMT: * n* = 407 HE-DMT: * n* = 211	Initial treatment with HE-DMT vs ME-DMT associated with a significant lower risk of SPMS conversion ( HR 0.66, 95% CI 0.44; 0.99, *p* = 0.046); 5-year absolute risk: 7% vs 12% Escalation from ME-DMT to HE-DMT within 5 years vs later associated with a significant lower risk of SPMS conversion ( HR 0.76, 95% CI 0.66; 0.88, *p* < 0.001; 5-year absolute risk: 8% vs 14%
He et al. [11]	Retrospective data from the MSBase registry and the Swedish MS registry with ≥ 6 years of follow-up	Early vs late HE-DMT start after clinical onset (0–2 vs 4–6 years) *HE-DMT: RTX, OCRE, MTX, ALEM, NAT*	Early HE-DMT: * n* = 213 Late HE-DMT: * n* = 253	Early vs late HE-DMT start was associated with a significantly lower EDSS score after 6 years (mean EDSS [standard deviation] = 2.2 [1.6] vs 2.9 [1.8], * p* < 0.001), which persisted throughout each year of follow-up until the 10th year after disease onset with a difference between groups of − 0.98 (95% CI − 1.51; − 0.45; * p* < 0.0001) across the 6–10 year follow-up period
Buron et al. [12]	Retrospective data from the Danish registry	First treatment: HE-DMT vs ME-DMT *ME-DMT: IFN-β, GA, TERI, DMF* *HE-DMT: FTY, NAT, CLAD, DAC, ALEM, OCRE*	ME-DMT: * n* = 194 HE-DMT: * n* = 194	At 4-year follow-up, HE-DMT vs ME-DMT associated with a significantly lower probability of a 6-month confirmed EDSS score worsening (16.7% [95% CI 10.4%; 23.0%] vs 30.1% [95% CI 23.1%; 37.1%]; HR 0.53 [95% CI 0.33; 0.83], *p* = 0.006)
Spelman et al. [13]	Retrospective data from the Danish and Swedish national MS registries	Swedish vs Danish cohorts *ME-DMT: IFN-β, GA, TERI, DMF* *HE-DMT: FTY, NAT, RTX, ALEM, OCRE*	Danish cohort: * n* = 1994 (92.3%) ME-DMT, *n* = 165 (7.7%) HE-DMT Swedish cohort: * n* = 1769 (65.5%) ME-DMT, * n* = 931 (34.5%) HE-DMT	The Swedish vs Danish treatment strategy associated with 29% in the rate of 24-week confirmed disability worsening ( HR 0.71 [95% CI 0.57; 0.90], *p* = 0.004) 24% in the rate of reaching an EDSS score ≥ 3.0 ( HR 0.76 [95% CI 0.60; 0.90], *p* = 0.03) 25% in the rate of reaching an EDSS score ≥ 4.0 ( HR 0.75 [95% CI 0.61; 0.96], *p* = 0.01)
Uher et al. [14]	Retrospective analyses from Avonex-Steroids-Azathioprine (*n* = 166), Study of Early IFN-β1a Treatment (*n* = 180), and in a cohort from the quantitative MRI project (*n* = 1557)	Escalation from ME-DMT to HE-DMT *ME-DMT: IFN-β, GA, TERI, DMF* *HE-DMT: FTY, NAT, MTX*	*n* = 94 (609 MRI scan)	BVL rates substantially decreased following treatment escalation (before: mean = 0.45, 95% CI − 0.54; − 0.37 vs after: mean = − 0.10, 95% CI − 0.13; − 0.07). Such differences were confirmed in adjusted mixed models, where treatment escalation resulted in a mean reduction of the BVL rate by 0.29% (*β* = − 0.29, 95% CI − 0.40; − 0.19, *p* < 0.001) Effects were only measurable two years after escalation to a HE-DMT
Iaffaldano et al. [60]	Retrospective analyses from the Italian MS Registry	EIT vs ESC *ESC: IFN-β, GA, DMF, FTY, TERI, AZA* *EIT: FTY, NAT, MTX, ALEM, OCRE, CLAD*	EIT: * n* = 364 ESC = *n* = 364	EIT vs ESC showed significantly lower mean annual EDSS changes (*p* < 0.02), with the differences in mean EDSS changes increasing from 0.10 (95% CI 0.01; 0.19, *p* = 0.03) at 1 year to 0.30 (95% CI 0.07; 0.53, *p* = 0.009) at 5 years and to 0.67 (95% CI 0.31; 1.03, * p* = 0.0003) at 10 years
Hanninen et al. [15]	Retrospective data from the Finnish registry	First treatment: HE-DMT vs ME-DMT *ME-DMT: IFN-β, GA, TERI, DMF* *HE-DMT: NAT, ALEM, OCRE, RTX*	ME-DMT: * n* = 1771 HE-DMT: * n* = 154	HE-DMT vs ME-DMT associated with a significantly lower probability of a 6-month confirmed EDSS score worsening (28.4% [95% CI 15.7; 39.3] vs 47.0% [95% CI 33.1; 58.1], *p* = 0.013)

*ALEM* alemtuzumab, *AZA* azathioprine, *BVL* brain volume loss, *CI* confidence interval, *CLAD* cladribine, *DAC* daclizumab, *DMF* dimethyl fumarate, *EDSS* Expanded Disability Status Scale, *EIT* early intensive treatment, *ESC* escalation, *FTY* fingolimod, GA glatiramer acetate, *HE*-*DMT* high-efficacy disease-modifying therapy, *HR* hazard ratio, *IFN*-*β* interferon beta, *ME*-*DMT* moderate-efficacy disease-modifying therapy, *MRI* magnetic resonance imaging, *MTX* mitoxantrone, *NAT* natalizumab, *OCRE* ocrelizumab, *RTX* rituximab, *TERI* teriflunomide

In a retrospective study from the MSBase registry and the Swedish MS registry with MS patients having at least 6 years of clinical follow-up [11], early vs late HE-DMT start, defined as between 0–2 or 4–6 years from clinical onset, respectively, was associated with a significantly lower EDSS score after 6 years (mean EDSS [standard deviation (SD)] = 2.2 [1.6] vs 2.9 [1.8], *p* < 0.001), which persisted throughout each year of follow-up until the 10th year after disease onset with a difference between groups of − 0.98 (95% confidence interval [CI] = − 1.51; − 0.45; *p* < 0.0001) across the 6–10 year follow-up period.

Similarly, a retrospective study from the Danish registry[12] showed that treatment initiation with HE-DMT compared to ME-DMT was associated with a significantly lower probability of a 6-month confirmed EDSS score worsening after 4 years of follow-up (16.7% [95% CI 10.4%; 23.0%] vs 30.1% [95% CI 23.1%; 37.1%]; HR 0.53, 95% CI 0.33; 0.83, *p* = 0.006).

Consistently, another recent retrospective study from the Finnish registry [15] showed that having HE-DMT instead of ME-DMT as the first treatment was associated with a significantly lower probability of a 6-month confirmed EDSS score worsening (28.4% [95% CI 15.7; 39.3] vs 47.0% [95% CI 33.1; 58.1], *p* = 0.013).

Finally, a study compared retrospectively two large cohorts from the Danish and Swedish National MS registries, which were characterized by different proportions of MS patients receiving HE-DMTs (Swedish = 65.5%; Danish = 7.7%) [13]. Interestingly, the study demonstrated indirectly that HE-DMTs as the first treatment were more effective since, compared to the Danish approach, the Swedish treatment strategy, being characterized by a larger use of HE-DMTs, was associated with a reduction of 29% in the rate of 24-week confirmed disability worsening (HR 0.71 [95% CI 0.57; 0.90], *p* = 0.004), of 24% in the rate of reaching an EDSS score ≥ 3.0 (HR 0.76 [95% CI 0.60; 0.90], *p* = 0.03), and of 25% in the rate of reaching an EDSS score ≥ 4.0 (HR 0.75 [95% CI 0.61; 0.96], *p* = 0.01).

In another retrospective study from the MSBase registry [10], compared to ME-DMT, initial treatment with HE-DMT was found to be associated with a significant lower risk of evolution to secondary progressive MS (hazard ratio [HR] 0.66, 95% CI 0.44; 0.99, *p* = 0.046, 5-year absolute risk = 7% vs 12%).

Recent studies also supported the superiority of HE-DMTs compared to an escalation approach. In a recent retrospective monocentric study [9], treatment with natalizumab or alemtuzumab was associated with a significantly lower 5-year change in EDSS compared to an escalation strategy (mean [standard deviation, SD] = 0.3 [1.5] vs 1.2 [1.5], *p* < 0.001), that remained significant after adjustment for relevant covariates (*β* = − 0.85; 95% CI − 1.38; − 0.32, *p* = 0.002). Moreover, median time to sustained accumulation of disability was significantly higher for HE-DMTs compared to the escalation strategy (6.0 [95% CI 3.17; 9.16] years vs 3.14 (95% CI 2.77; 4.00) years, respectively, *p* = 0.05).

Similarly, in another retrospective study from the Italian MS registry [60], compared to the escalation strategy, MS patients treated with HE-DMT showed significantly lower mean annual EDSS changes (*p* < 0.02), with the differences in mean EDSS changes increasing from 0.10 (95% CI 0.01; 0.19, *p* = 0.03) at 1 year to 0.30 (95% CI 0.07; 0.53, *p* = 0.009) at 5 years and to 0.67 (95% CI 0.31; 1.03, *p* = 0.0003) at 10 years.

Moreover, escalation from ME-DMT to HE-DMT within 5 years vs later was also found to be associated with a significant lower risk of SPMS conversion (HR 0.76, 95% CI 0.66; 0.88, *p* < 0.001; 5-year absolute risk: 8% vs 14%) [10].

Finally, another retrospective study[14] demonstrated the efficacy of HE-DMTs in reducing neurodegenerative processes, quantified by brain volume loss. In particular, rates of brain volume loss substantially decreased following treatment escalation (see Table 2) from ME-DMT to HE-DMT (before: mean = − 0.45 [95% CI − 0.54; − 0.37] vs after: mean = − 0.10 [95% CI − 0.13; − 0.07]). Such differences were confirmed in adjusted mixed models, where treatment escalation resulted in significant mean reduction of brain volume loss rate by 0.29% (*β* = − 0.29, 95% CI − 0.40; − 0.19, *p* < 0.001). Of note, effects were measurable at least two years after escalation to an HE-DMT, thus supporting the importance of not wasting time to limit neurodegeneration.

## Treatment strategies based on patients’ profiles

Tailored treatment is ideal for MS patients due to the heterogeneity of MS clinical manifestations, severity and long-term evolution and it is currently more feasible thanks to the availability of a large spectrum of different DMTs.

Personalized treatment selection for each individual MS patient is typically influenced by many factors, including demographic, environmental, clinical characteristics and prognostic factors, currently available guidelines, treatment burden and costs and patients’ choice [61].

Current evidence [9–15, 61] and treatment guidelines [4, 5] suggest that early use of HE-DMTs may represent the appropriate therapeutic approach in the presence, already from the earliest phases of MS, of negative prognostic factors being associated with long-term disease progression (Table 3) [11, 62–64].

**Table 3:** Summary of the main negative prognostic factors predicting disability progression in MS patients

*Predictors*
*Patient’s demographics and environmental factors*
Non-Caucasian
Older age
Male sex
Obesity (particularly in childhood and adolescence)
Smoking
*Clinical factors*
Onset with motor, cerebellar, or bladder/bowel symptoms
Multifocal onset (≥ 2 functional systems involved simultaneously)
Higher relapse rate in the first 2–5 years from disease onset
Short inter-attack latency
Incomplete recovery after a relapse
Severe clinical relapses
Higher disability accumulation in the first 2–5 years from disease onset
Continued disease activity despite DMT
Shorter time to conversion to SPMS
Cognitive impairment
*Biochemical factors*
Presence of cerebrospinal OCBs
High NfL level
*Neuroradiological factors*
Higher number and volume of T2-hyperintense lesions
Brainstem and cerebellar lesions
Spinal cord lesions (especially affecting the central GM)
T1-hypointense lesions (“black-holes”)
Cortical lesions
Presence of gadolinium-enhancing lesions
New T2 lesions formation in the first 5 years
Chronic active lesions (paramagnetic iron rim or slowly expanding)
Brain atrophy (especially GM)
Spinal cord atrophy (especially GM)

*GM* gray matter; *NfL* neurofilament light chain, *OCB* oligoclonal bands, *SPMS* secondary progressive multiple sclerosis

Conversely, escalation strategies with the use of ME-DMTs are still often preferred for MS patients with the evidence of a milder disease course, because of their superior safety profile and lower burden for both MS patients and clinicians. Moreover, the identification of prognostic factors can be challenging and current knowledge gaps, including validation of biomarkers and treatment algorithms, may limit their implementation in the clinical setting.

Taking into account the emerging evidence regarding the beneficial effect or early HE-DMT start, beside the early use of an HE-DMT in case of bad prognostic factors suggestive of an aggressive disease, it may be rewarding to offer an early treatment with an HE-DMT also to MS patients with moderate activity. Clearly, the selection of the best treatment option should also be based on specific patient-related factors beyond the clinical prognosis and on a shared decision-making between the MS patient and the clinician.

A careful anamnestic patient’s medical history is necessary to identify possible comorbidities that may represent relative or absolute contraindications for specific DMTs (e.g., cardiac or cerebrovascular disease for sphingosine 1-phosphate receptor modulators, progressive multifocal leukoencephalopathy for natalizumab, malignancies for immunosuppressants or immunodepleting therapies, mood disorders and thyroid dysfunctions for interferon betas, other dysimmune disorders for alemtuzumab, etc.).

Moreover, MS preferentially affects young women aged 20–40 years, thus it is advisable to discuss possible pregnancy plans and to follow specific recommendations since several DMTs are contraindicated during pregnancy and should be interrupted before trying to conceive [65].

Finally, offering to MS patients the possibility to express their preference and choice regarding many aspects of DMTs (route of administration, posology, concerns regarding specific side effects and safety issues, frequency of monitoring, etc.) may positively influence adherence to treatments.

## Overcoming barriers to HE-DMTs’ early use

Equal access to care should be guaranteed to all MS patients, allowing access to highly specialized MS Centers with experienced clinicians to receive an appropriate treatment.

Despite this, several limitations still hamper their use especially in the earliest phases of the disease.

The perception of an unfavorable risk/benefit ratio in the long term of HE-DMTs compared to ME-DMTs, due to safety concerns, especially over the long term (i.e., risk of chronic immunosuppression, infections and malignancies), is one of the most relevant limitations for the decision to use HE-DMTs only for more aggressive and severe forms of MS.

However, the long-term risk/benefit ratio of HE-DMTs is likely to be more favorable if these drugs are started early during the MS course. Their early use in younger MS patients may optimize the therapeutic window for potent drugs to exert their strong anti-inflammatory action against a highly active and pro-inflammatory immune system. Conversely, the beneficial effects of HE-DMTs may be more limited in MS patients with older age, longer disease duration, more severe CNS damage and disability, where the immuno-senescence occurring with aging may substantially reduce the therapeutic effects of HE-DMTs but also increase the risk of infections and malignancies [66]. However, further studies are still needed to explore the effects and safety of HE-DMTs in older MS patients.

Although long-term safety data are still lacking, especially for recently introduced HE-DMTs, available results from long-term follow-up studies of MS patients with HE-DMTs seem to suggest that the safety profiles of HE-DMTs do not substantially differ from ME-DMTs [67–72]. Accordingly, it does not seem very reasonable to delay HE-DMT use even for a marginally increased risk of infections and/or malignancies considering the beneficial effects against MS-related neurodegenerative processes and disability progression that would be lost with a more conservative strategy.

Moreover, the escalation approach may be characterized by a sequential use of different DMTs with heterogeneous immunomodulatory/immunosuppressive/depleting actions that may determine complex and variable effects on the immune systems whose long-term consequences may be much more unpredictable compared to a more selective and targeted action of a single HE-DMT started early and continued for a longer period. Accordingly, it is necessary to weight the relative safety risks associated with the early use of HE-DMTs with those of worse disease progression with alternative treatment approaches that are safer.

Other barriers to the early use of HE-DMTs include challenges in the management of treatment start and monitoring, MS patients’ preferences, restricted access to HE-DMTs according to guidelines and regulatory rules, and sustainability [4, 5, 20, 61, 73, 74]. However, safety issues and the other aforementioned barriers do not apply to each HE-DMT and none of these appear insuperable.

## Conclusions

The therapeutic goals of MS treatment are moving towards the prevention of CNS neurodegeneration and irreversible disability that are driven by heterogeneous pathological processes that begin from the clinical onset of the disease and that seem to occur mainly independently from overt relapse activity.

To this aim, there is an urgent need to identify the best therapeutic approach to prevent MS progression. Emerging evidence suggests that the early use of HE-DMTs represents a rewarding strategy with the most positive benefit/risk ratio since early HE-DMT use is associated with a significant greater reduction not only of inflammatory activity, but also of disease progression compared to delayed HE-DMT or escalation strategy and seems to be associated with acceptable long-term safety risks.

Barriers to an early use of HE-DMTs are still present and include concerns for long-term safety, challenges in the management of treatment start and monitoring, MS patients’ preferences, restricted access to HE-DMTs according to guidelines and regulatory rules, and sustainability. However, these barriers do not apply to each HE-DMT and none of these appear insuperable.

Further demonstrations of the efficacy of the early use of HE-DMTs on more sensitive and specific clinical, neuroradiological and biological outcomes, together with the collection of long-term safety data, are likely to generate greater confidence and agreements on the benefit/risk profile over time of such an approach, thus promoting updates in guidelines of MS treatment algorithms and the removal of restrictions to HE-DMT access for MS patients, especially in the earliest phases of the disease.
