# Correction to: Early use of high-efficacy disease-modifying therapies makes the difference in people with multiple sclerosis: an expert opinion

**DOI:** 10.1007/s00415-022-11385-4

**Published:** 2022-09-22

**Authors:** Massimo Filippi, Maria Pia Amato, Diego Centonze, Paolo Gallo, Claudio Gasperini, Matilde Inglese, Francesco Patti, Carlo Pozzilli, Paolo Preziosa, Maria Trojano

**Affiliations:** 1 Neuroimaging Research Unit, Division of Neuroscience, IRCCS San Raffaele Scientific Institute, Via Olgettina, 60, 20132 Milan, Italy; 2 Neurology Unit, IRCCS San Raffaele Scientific Institute, Via Olgettina, 60, 20132 Milan, Italy; 3 Neurorehabilitation Unit, IRCCS San Raffaele Scientific Institute, Via Olgettina, 60, 20132 Milan, Italy; 4 Neurophysiology Service, IRCCS San Raffaele Scientific Institute, Via Olgettina, 60, 20132 Milan, Italy; 5 Vita-Salute San Raffaele University, Milan, Italy; 6 Department NEUROFARBA, University of Florence, Florence, Italy; 7 IRCCS Fondazione Don Carlo Gnocchi, Florence, Italy; 8 Department of Systems Medicine, Tor Vergata University, Rome, Italy; 9 Unit of Neurology, IRCCS Neuromed, Pozzilli, IS Italy; 10 Department of Neuroscience, University of Padova, Padua, Italy; 11 Department of Neurosciences, S Camillo Forlanini Hospital Rome, Rome, Italy; 12 Department of Neuroscience, Rehabilitation, Ophthalmology, Genetics, Maternal and Child Health (DINOGMI), University of Genoa, Genoa, Italy; 13 IRCCS Ospedale Policlinico San Martino, Genoa, Italy; 14 Department GF Ingrassia, Medical, Surgical Science and Advanced Technologies, University of Catania, Catania, Italy; 15 Center for Multiple Sclerosis, Policlinico “G Rodolico”, University of Catania, Catania, Italy; 16 S. Andrea MS Center, Sapienza University, Rome, Italy; 17 Department of Basic Medical Sciences, Neuroscience, and Sense Organs, University of Bari “Aldo Moro”, Bari, Italy

## Correction to: Journal of Neurology y (2022) 269:5382–5394 10.1007/s00415-022-11193-w

The article Early use of high-efficacy disease-modifying therapies makes the difference in people with multiple sclerosis: an expert opinion, written by Massimo Filippi, Maria Pia Amato, Diego Centonze, Paolo Gallo, Claudio Gasperini, Matilde Inglese, Francesco Patti, Carlo Pozzilli, Paolo Preziosa and Maria Trojano, was originally published Online First without Open Access. After publication in volume 269, issue 10, pages 5382–5394 the author decided to opt for Open Choice and to make the article an Open Access publication. Therefore, the copyright of the article has been changed to © The Author(s) 2022 and this article is licensed under a Creative Commons Attribution 4.0 International License, which permits use, sharing, adaptation, distribution and reproduction in any medium or format, as long as you give appropriate credit to the original author(s) and the source, provide a link to the Creative Commons licence, and indicate if changes were made. The images or other third party material in this article are included in the article’s Creative Commons licence, unless indicated otherwise in a credit line to the material. If material is not included in the article’s Creative Commons licence and your intended use is not permitted by statutory regulation or exceeds the permitted use, you will need to obtain permission directly from the copyright holder. To view a copy of this licence, visit http://creativecommons.org/licenses/by/4.0/.

The original article has been corrected.

